# Transcatheter Aortic Valve Replacement in Bicuspid Aortic Valve Disease: A Review of the Existing Literature

**DOI:** 10.7759/cureus.78192

**Published:** 2025-01-29

**Authors:** Chmsalddin Alkhas, George G Kidess, Matthew T Brennan, Jawad Basit, Farah Yasmin, Wael Jaroudi, M. Chadi Alraies

**Affiliations:** 1 Department of Internal Medicine, Wayne State University Detroit Medical Center, Detroit, USA; 2 Department of Medicine, Wayne State University School of Medicine, Detroit, USA; 3 Department of Surgery, Holy Family Hospital, Rawalpindi, PAK; 4 Department of Cardiology, Rawalpindi Medical University, Rawalpindi, PAK; 5 Department of Internal Medicine, Yale School of Medicine, New Haven, USA; 6 Department of Cardiovascular Medicine, Clemenceau Medical Center, Beirut, LBN; 7 Department of Cardiology, Wayne State University Detroit Medical Center, Detroit, USA

**Keywords:** bicuspid aortic valve disease, transcatheter aortic replacement, transcatheter aortic valve implant, transcatheter aortic valve implantation (tavi), vulvular heart disease

## Abstract

Transcatheter aortic valve replacement (TAVR) is a minimally invasive procedure used to replace a damaged aortic valve with a prosthetic valve. TAVR has exceeded surgical aortic valve replacement (SAVR) due to shorter procedures and recovery times. Though initially approved for patients with aortic stenosis at a high surgical risk, TAVR’s indications have now broadened to include high, intermediate, and low-risk patients. This review focuses on the evolving role of TAVR in patients with bicuspid aortic valves (BAV). We examine the anatomical and hemodynamic differences between tricuspid aortic valve and BAV, highlighting the unique challenges TAVR faces in BAV patients

## Introduction and background

In 2019, for the first time in the United States, the number of transcatheter aortic valve replacement (TAVR) procedures exceeded surgical aortic valve replacement (SAVR) procedures [1]. TAVR was FDA-approved in 2011 for severe symptomatic aortic stenosis (AS) patients who are at extremely high risk of undergoing SAVR [1]. From there, the approval was expanded to include high, intermediate, and, lastly, low-risk patients. This overtake is owed to the persistent development of TAVR technology that improved the safety and efficiency in AS patients. This led to an increase in the indications to undergo TAVR procedures, which eventually caused TAVR to exceed SAVR [1]. Whether for bicuspid aortic valve (BAV) or tricuspid aortic valve (TAV), aortic valve replacement is the definitive treatment option for symptomatic and/or severe AS patients. In this review, we will go through the latest indications and developments in the TAVR procedure, specifically in BAV patients. In addition, we will discuss patient outcomes, safety, and the conditions that favor TAVR over SAVR in BAV patients.

## Review

Normal and bicuspid aortic valve anatomy and hemodynamics

**Figure 1:**
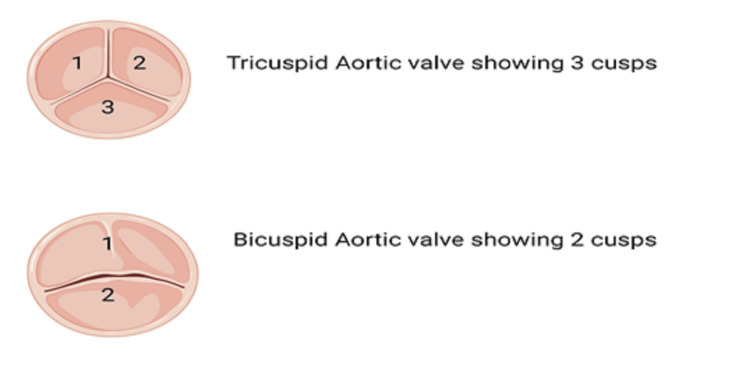
Tricuspid and bicuspid aortic valves Image Credit: Chmsalddin Alkhas

The aortic valve is a part of the aortic root that separates the left ventricle outflow tract from the ascending aorta [2]. The aortic valve is made of three semilunar cusps or leaflets as shown in Figure 1, each forming a pouch called sinus of Valsalva, these sinuses are anatomically named based on their proximity to the coronary ostia if present (left, right, non-coronary cusp) [3-5].

**Figure 2:**
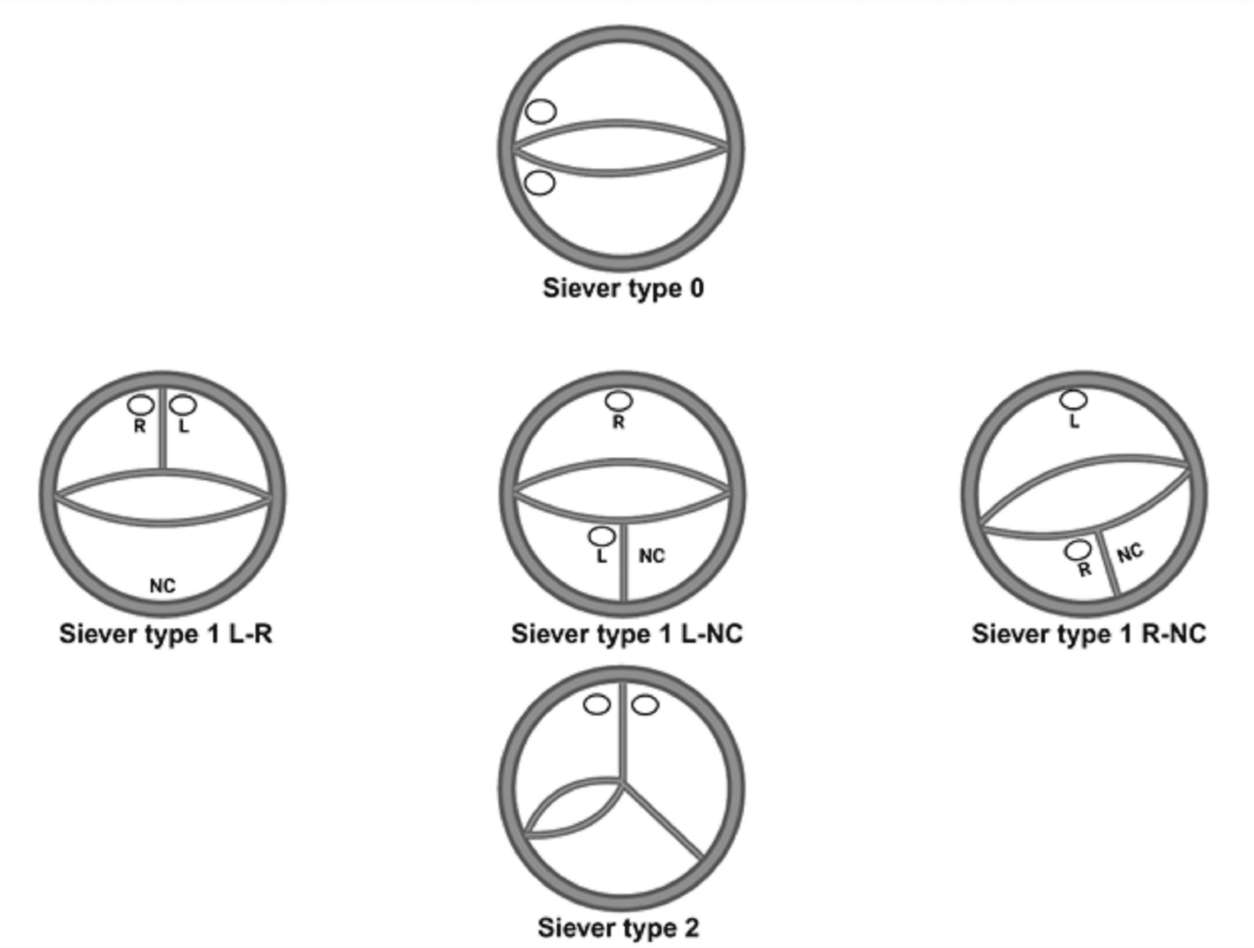
Siever classification for BAV based on the number of raphes. BAX: bicuspid aortic valve Image Credit: Chmsalddin Alkhas

When the leaflets meet, they form commissures that are attached to the aortic wall. On the contrary, BAV has two cusps, one of which has a thin layer called raphe that divides one of the cusps [4]. The most widely used morphological classification of BAV classifies the valve according to the number of raphes present to either siever 0, 1, or 2 as in Figure 2 [6]. 

BAV shows aberrant hemodynamic patterns during the cardiac cycle in comparison to TAV, especially during systole [7]. Blood flow in BAV is decentralized from the center of the aortic lumen and follows an intense spiral shape of flow that causes more stress to the aortic wall. All of the previous limit the valve's ability in the long run and it begins to deteriorate in functionality later in life, either in adolescence or adulthood [4]. BAV is known to be the most common congenital heart disease with a 1% incidence rate in the population and a prevalence of 0.4-2.25% [8].

BAV: a precursor to valvular and aortic complications

BAV patients are at risk of developing several valvular-related conditions in their lifetime [9]. Of these, the most common and serious conditions are valvulopathies (aortic regurgitation and AS) and aortic wall structural alterations like ascending aortic dilatation and aneurysms [3,4,7,10]. Aortic valve calcification leading to AS occurs at a faster rate, about one decade earlier in BAV patients than in individuals with tricuspid aortic valves [4,11]. Of BAV patients who developed heart failure, 51% had one of the mentioned valvulopathies [10]. Even if it is to a lower extent, it is worth noting that BAV patients are at risk of developing infective endocarditis and aortic dissection [12]. The definitive treatment for BAV valvular-related conditions is TAVR or SAVR; indications and differences in both strategies will be discussed further in this review. Strict blood pressure control in BAV patients is crucial to maintaining the valve's integrity and the ascending aorta [13]. American College of Cardiology (ACC) and American Heart Association (AHA) recommend treating hypertension in BAV patients according to standard guideline-directed medical therapy and using statins for stenotic calcified valves [14]. Although there have been hypotheses regarding a possible reduction of ascending aortic dilatation in patients with BAV by beta-blockers or angiotensin-converting enzyme (ACE) inhibitors, current clinical evidence does not support a causal association between these drugs and a decreased rate of aortic dilatation [15,16].

TAVR procedure

TAVR is a minimally invasive procedure that has been utilized to treat patients with aortic valvular disease and high surgical risk [17]. The most common approach for TAVR is endovascular through a femoral arterial access site preferably through non-diseased arteries, although newer systems utilize smaller sheaths accommodating arterial diameters greater than 6-8 mm depending on the sheath size. Much less commonly in the case of severely diseased femoral arteries, surgical approaches such as retroperitoneal, axillary, and transapical approaches have been utilized [18]. Types of valves utilized in TAVR are grouped into either self-expanding such as the Edwards SAPIEN (Edwards Lifesciences Corporation, Irvine, California, United States), or balloon-expandable valves such as the CoreValve Revalving system (Medtronic plc, Minneapolis, Minnesota, United States).

Landmark trials for both types of valves including the Placement of Aortic Transcatheter Valves (PARTNER) (NCT00530894) and CoreValve US Pivotal (NCT01240902) trials have shown lower mortality rates in either kind of TAVR device compared to SAVR [17]. Recent studies show no difference in mortality or complication rates between balloon-expandable or self-expanding valves, although self-expanding valves have had higher device success rates [17,19-23].

TAVR vs. SAVR: weighing the benefits and risks

Several trials have compared the performance of TAVR to SAVR. Both the PARTNER and Nordic Aortic Valve Intervention (NOTION) trials showed no difference in mortality or stroke between TAVR and SAVR, with the NOTION trial also showing lower rates of bleeding, cardiogenic shock, acute kidney injury, and new-onset or worsening atrial fibrillation [24,25]. Importantly, TAVR is associated with reduced recovery times and in some cases improved functionality than SAVR [17,26]. Various studies have shown improved quality of life indices in patients undergoing TAVR compared to SAVR, especially in patients with worse baseline functionality, as well as a less stressful physical and mental recovery experience [17,27-29]. One of the disadvantages of TAVR compared to SAVR is its durability, with early studies showing higher structural valve deterioration (SVD) and bioprosthetic valve failure (BVF) after five years in TAVR patients [30]. More recent studies have shown either similar or better outcomes for SVD and BVF in TAVR patients in up to eight years; however, due to lack of long-term data, SAVR remains the recommended approach in patients younger than 65 years [31-33]. Another consideration is patient-prosthetic matching with regard to annulus size to avoid adverse outcomes such as paravalvular regurgitation [18]. Recent studies suggest that TAVR has similar outcomes with regards to patient-prosthesis matching as SAVR despite higher rates of paravalvular regurgitation, with one study showing balloon-expandable valves having higher rates of patient-prosthesis mismatch than self-expandable valves [34-37].

TAVR limitations

Importantly, TAVR carries the risk of various complications with significant morbidity and mortality. One of its most feared complications is cerebrovascular ischemia, occurring in up to 6% of TAVR patients and mainly caused by manipulation and embolization of preexisting plaque [17,18]. The rate of TAVR-associated stroke has decreased significantly in more recent studies, mainly due to the use of smaller, less traumatic catheters and improved technique, and the development of new devices such as cerebral embolic protection devices has shown promise in preliminary studies [17,18]. Paravalvular regurgitation has also been shown to occur at high rates and is associated with increased mortality, although recent advancements in device development have reduced the rates of this complication [17,18]. Coronary artery occlusion is another serious complication of TAVR, especially after valve-in-valve TAVR, that occurs at higher rates than in SAVR and needs to be addressed by meticulous preoperative assessment [17,31]. Other common complications of TAVR include conduction abnormalities necessitating pacemaker implantation, acute kidney injury, bleeding, access-related complications, and valve thrombosis [18,31].

TAVR for BAVs: a breakthrough in lower-risk patients

TAVR was initially developed and validated for patients with TAVs. Traditionally, SAVR has been the preferred treatment for BAV patients according to international guidelines [38]. Nevertheless, in 2019, the FDA and the European Conformity approved TAVR to be indicated in lower-risk BAV patients [39]. This is because of the complex anatomy of BAV that may jeopardize the success of TAVR replacement and the fear of complications related to BAV. However, due to TAVR's effectiveness, safety, and minimally invasive profile, it has received significant attention over the last 15 years and led to its approval by the FDA for low-risk patient categories [39].

Complex BAV anatomy and fear of complications

The dimensions of all components of the aortic valve complex are generally larger in BAV than in the TAV, increasing the likelihood of having an annulus size outside of the range covered by the currently available transcatheter heart valve [39]. In addition, about two-thirds of BAV patients have a non-tubular aortic valve structure, defined by either a flared or tapered shape [40]. BAVs are significantly more calcified than TAVs [41], and it was found that BAVs are heavier in weight and their calcium score exceeded TAVs [40], raising the possibility of erroneous prosthesis deployment or paravalvular leak during TAVR operations. Also, patients with BAVs, in particular the ones with raphe [42], are at increased risk of aortopathies such as aortic root or ascending aortic dilatation which may compromise the efficacy of TAVR. The complexities mentioned above have contributed to apprehension regarding procedural outcomes, leading to the exclusion of BAV patients in the randomized controlled trials evaluating TAVR efficacy [39,43]. 

Important developments propelling TAVR's extension to BAV patients

The expanding spectrum of TAVR indications is a result of technological advancements in valve design, the evolution in imaging modalities, and accumulating evidence from clinical trials and observational studies, including promising outcomes in BAV patient cohorts. Initially, in BAV patients, TAVR procedure utilized first-generation ballon-expandable valves, a couple of years later, a second generation of self-expandable and ballon-expandable valves were used. Both valve generations demonstrated substantial rates of complications, notably paravalvular leak, and pacemaker implantation [44]. The third generation of valves is SAPIEN 3 which incorporates an outer layer of seal to prevent or minimize paravalvular leak and it succeeded in minimizing it [45-57]. SAPIEN 3 Ultra Resilia valve (S3UR) symbolizes the newest balloon-expandable valve engineered to lower valve calcification that leads to functional deterioration [48]. S3UR showed lower mean aortic gradients and paravalvular leaks than previous generations of balloon-expandable valves [48]. Valve types and sizing are areas in which precision plays a big role in determining TAVR outcomes and complications, if the valve is over the annulus size; the patient is at risk of conduction abnormalities placing him in need of a permanent pacemaker, on the other side; a smaller size valve increases the risk of paravalvular regurgitation [49]. The precise three-dimensional (3D) measurements of the aortic root dimensions using multi-detector CT (MDCT) or transoesophageal echocardiogram (TOE) imaging have shown to be extremely helpful in lowering the incidence of paravalvular leaks and better TAVR outcomes [39]. It replaced transthoracic echo and became the gold standard for TAVR pre-procedural assessment [49].

TAVR’s evolvement of indications and level of safety in BAV

SAVR was and still is the gold standard for BAV-related aortopathies. TAVR procedure came to the game as a solution for AS patients who are considered high-risk and could not undergo SAVR [50,51]. In this category of patients, TAVR showed similar survival rates [52] or even better [53] compared to SAVR at one year proving it is non-inferior to SAVR concerning short-term safety. TAVR has also been shown to be non-inferior to SAVR in intermediate-risk AS patients at 24 months of the procedure [54]. That led the AHA\ACC in 2020 to consider TAVR as the preferred treatment option in high-risk (>65 years) patients with severe AS [55]. The challenge now is to find out whether TAVR is suitable for low-risk and young patients. According to the 2020 ACC/AHA guidelines, SAVR remains the preferred treatment option for low-risk severe AS patients [55]. However, this may change in the future considering the growing research on TAVR and the constant development in improving its efficacy and lowering its complications. Several studies demonstrated a promising future for TAVR when compared to SAVR with regard to safety and efficacy, however, TAVR is still showing higher rates of permanent pacemaker implantation (PPI) and paravalvular regurgitation (PVR), which remain significant challenges in the field of TAVR [24,55,56]. In addition, TAVR still lacks long-term follow-up in this category of patients and has not been tested in patients younger than 65 years.

BAV and TAVR: a complex relationship

Nowadays, only a small percentage (3.4%) of TAVR patients have BAV [58], as the ACC/AHA considers TAVR a viable alternative to SAVR in BAV patients suffering from AS [55]. This is because of many considerations: patients are relatively younger age than tricuspid AS patients, earlier studies using older generations of valves suggested PVR higher in BAV patients undergoing TAVR [59], and as mentioned before, BAV patients were excluded from the randomized controlled trials (RCTs), necessitating the need for future RCTs in this patient category, especially after new valve generations showed better outcomes in terms of PVR [60]. Also, it was found that TAVR in BAV was more associated with stroke than in TAV patients [50].

What predicts TAVR's success in BAV and who is a suitable candidate?

The amount of calcification, whether on the valve leaflets or raphe, is now considered predictive regarding TAVR outcomes in BAV patients. Also, certain valve morphologies are associated with an increased risk of complications. Yoon et al. found that BAV patients who had excess raphe and leaflet calcification and underwent TAVR were associated with more complications like stroke and PVR [61]. In addition, patients who show significant calcification should be deferred from undergoing TAVR. BAV patients with BAV morphologies showing Type 1 N-L and Type 2 L-R/R-N are more prone to PVR [62]. Table 1 shows conditions that may lead physicians to choose TAVR or SAVR in BAV patients.

**Table 1:** Conditions that guide the choice of TAVR vs SAVR in patients with BAV TAVR: transcatheter aortic valve replacement; SAVR: surgical aortic valve replacement; BAV: bicuspid aortic valve

	TAVR	SAVR
Siever type	Type 1	Type 1 N-L, Type 2 L-R/R-N
Raphe and leaflet Calcification	Uniform moderate calcification	Heavily calcified
Concomitant aortopathy	Absent	Aortopathy >4.5 cm^50^
Age	> 70 years	<70 years
Isolated Aortic regurge	Absent	Present

Comparison of TAVR vs SAVR in BAV stenosis

Complication Rates

No prospective RCT has been performed comparing TAVR to SAVR for the treatment of BAV stenosis. In the absence of such a study, meta-analyses have pooled retrospective studies to yield a higher-powered comparison. One meta-analysis comparing 6,550 patients across six studies found no significant increase in in-hospital mortality in TAVR over SAVR (OR 1.11; p = 0.75) [63]. Another meta-analysis of 62,981 patients found no difference in three-day mortality [64]. Studies have also found no difference in short-term, 30-day mortality between SAVR and TAVR treatment groups [65,66]. A retrospective analysis of 1023 patients with bicuspid aortic valves found no significant difference in two-year mortality between the TAVR (9.7%) and SAVR (18.7%) groups (p=0.268) [65]. Notably, the risks of major complications such as myocardial ischemia and stroke were comparable between SAVR and TAVR patients [63,66-68]. However, other complications varied between the two groups. For instance, TAVR patients were more likely than SAVR patients to require a post-procedure pacemaker implant [63,64,66]. This is consistent with studies showing higher rates of post-procedure pacemakers in patients undergoing TAVR with tricuspid valve anatomy [68,69]. On the other hand, bleeding events, acute kidney injuries (AKIs), and respiratory complications were more common in SAVR patients [62,66]. SAVR patients also experienced a longer length of stay compared to those who underwent TAVR [63].

Post-Procedural Valve Hemodynamics

Overall, the transvalvular gradients in BAV patients did not significantly differ in TAVR versus SAVR groups [64]. Studies comparing TAVR in BAV versus TAV patients supported these findings, noting no significant difference in valve gradients between the two patient populations [70,47]. However, studies did show higher rates of paravalvular leaks in patients with BAVs who received TAVR, compared to SAVR [63,65]. The anatomy of BAVs may play a role in the risk of developing paravalvular leaks. Husso et al. used the Sievers BAV classification system to determine that patients with type 1 N-L and type 2 L-R/R-N experienced higher rates of mild-to-severe leaks compared to other anatomies [65]. Zito et al. also noted the challenge of TAVR when treating type 1 bicuspid aortic valves, resulting in a 4% risk of moderate to severe paravalvular leaks [71]. New technology may help to reduce these rates. One sub-analysis of TAVR procedures with newer-generation devices found a statistically significant difference in paravalvular leaks that disappeared [65]. The 2020 ACC/AHA Guidelines support these findings, pointing out that data from the Society of Thoracic Surgeons (STS)/ACC Transcatheter Valve Therapies Registry showed no difference in paravalvular leaks between bicuspid and tricuspid aortic valve patients who received TAVR [72].

Patient-Specific Considerations

Age is a major difference between patients undergoing repair of BAV versus TAV. For example, a study by Lim et al. found moderate to severe AS present at an average age of 77.3 years in those with TAVs versus 55.3 years in those with BAVs [73]. Because of the early age of onset for patients with BAVs, the long-term viability of TAVR procedures must be studied. Another consideration is that BAVs are associated with higher rates of aortopathies compared to TAVs. Patients with BAVs experience higher rates of aortic aneurysms and dissections [74]. They are also prone to aortic valve regurgitation [75]. These aortic comorbid conditions increase the complexity of TAVR procedures and may necessitate other surgical interventions, making SAVR a more logical choice [76].

Anatomical Considerations

BAVs present unique anatomical challenges to performing a TAVR procedure. BAVs often involve asymmetric valve annuli that can lead to a non-uniform expansion of the TAVR device [77]. This has been shown to increase the risk of paravalvular regurgitation [63]. In addition, BAV patients may present with excess leaflet calcification, which results in higher rates of complications such as paravalvular regurgitation and aortic root injuries [61]. Patients with BAVs tend to have smaller left ventricular outflow tracts (LVOTs) and dilated aortas, further complicating the accurate placement of valves during TAVR procedures [78].

Patient-centered decision-making and future directions

The 2020 ACC/AHA Guideline for the Management of Patients with Valvular Heart Disease gives a Class 2b recommendation for considering TAVR as an alternative to SAVR in patients with BAVs after considering “patient-specific procedural risks, values, trade-offs, and preferences, and when the surgery is performed at a Comprehensive Valve Center [72]. The recommendation is backed by Level of Evidence B-NR, indicating a reliance on non-randomized trials. These guidelines acknowledged that although older TAVR procedures demonstrated higher rates of paravalvular leaks, newer-generation TAVR devices have eliminated this difference. The guidelines also stressed the need for long-term RCTs for BAV patients, to examine TAVR device durability in this younger patient population. Ultimately, the decision to consider a TAVR device for BAV repair must take a patient-driven approach. Individual patient characteristics, anatomies, and values will guide the discussion of whether TAVR or a SAVR procedure is the best route for each patient. Digital AS Severity Index (DASSi) is a biomarker calculated by a new artificial intelligence (AI)-based video technology that can predict the development of severe AS. This AI technology represents a promising advancement that might help cardiologists in identifying patients who may require aortic valve replacement and facilitate the early detection of AS. This video technology has proven to be effective in a cohort study in which faster peak aortic valve velocity was linked to a higher baseline DASSi [79]. Also, patients who had a DASSi score of 0.2 or more were at increased risk of requiring aortic valve replacement [79].

**Figure 3:**
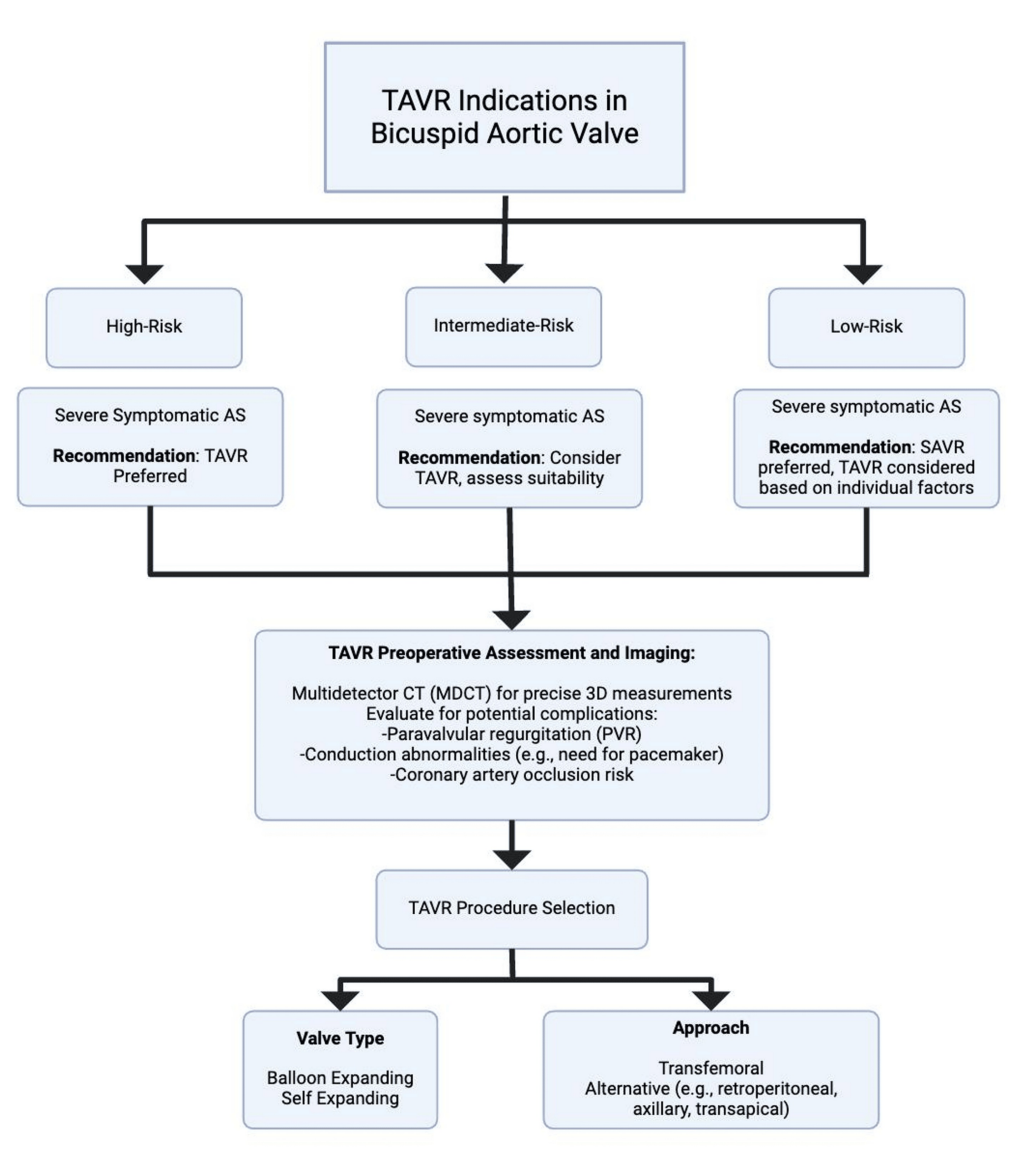
TAVR indications for patients with BAV Image Credit: George G. Kidess TAVR: transcatheter aortic valve replacement; BAV: bicuspid aortic valve; SAVR: surgical aortic valve replacement; AS: aortic stenosis

The advancement in TAVR valves and technology has mirrored the massive expansion in the field of interventional cardiology, with the development of newer generation valves leading to increased procedural success, enhanced performance, and reduced paravalvular leak [80]. Importantly, issues of bioprosthesis durability are also being addressed by devices such as a novel polymeric valve, which has been shown to meet the required hemodynamic parameters and have an estimated durability of over 20 years [81]. Specifically to BAV patients, we look forward to the results of various clinical trials such as the Medtronic low-Risk Bicuspid Study (NCT03635424), the HANGZHOU Solution in Bicuspid AS Undergoing TAVR (NCT04722796), Downsizing Strategy vs Standard Sizing Strategy TAVR in Bicuspid Aortic Stenosis (NCT05511792), and the Progression of Ascending Aorta Diameters in Bicuspid Aortic Valve After Transcatheter or Surgical Replacement (NCT05708118). The results of these studies may further clarify the efficacy and safety of TAVR in the BAV patient population. While issues such as post-TAVR pacemaker implantation and stroke need to continue being addressed, the improvements in TAVR technology and the inclusion of groups such as BAV patients are promising [80]. Figure 3 shows a summary of TAVR indications in BAVs.

## Conclusions

TAVR has emerged as a promising treatment for AS, particularly for high-risk patients, and has expanded its indications to include low-risk and BAV patients. Although initially contraindicated for BAV patients due to anatomical complexities, advancements in TAVR technology and improved valve designs have significantly reduced complications like paravalvular leaks and pacemaker implantation. Despite these improvements, challenges remain, particularly regarding long-term durability, stroke risk, and aortic root complications in BAV patients. As ongoing clinical trials and technological innovations continue to shape the field, TAVR's role in BAV treatment will likely evolve. Ultimately, patient-specific factors and careful decision-making will guide the choice between TAVR and SAVR, with future studies necessary to solidify long-term outcomes and enhance procedural safety.
